# Positive Effects of Plant Genotypic and Species Diversity on Anti-Herbivore Defenses in a Tropical Tree Species

**DOI:** 10.1371/journal.pone.0105438

**Published:** 2014-08-20

**Authors:** Xoaquín Moreira, Luis Abdala-Roberts, Víctor Parra-Tabla, Kailen A. Mooney

**Affiliations:** 1 Department of Ecology and Evolutionary Biology, University of California Irvine, Irvine, California, United States of America; 2 Departamento de Ecología Tropical, Campus de Ciencias Biológicas y Agropecuarias, Universidad Autónoma de Yucatán, Mérida, Yucatán, México; University of Oxford, United Kingdom

## Abstract

Despite increasing evidence that plant intra- and inter-specific diversity increases primary productivity, and that such effect may in turn cascade up to influence herbivores, there is little information about plant diversity effects on plant anti-herbivore defenses, the relative importance of different sources of plant diversity, and the mechanisms for such effects. For example, increased plant growth at high diversity may lead to reduced investment in defenses via growth-defense trade-offs. Alternatively, positive effects of plant diversity on plant growth may lead to increased herbivore abundance which in turn leads to a greater investment in plant defenses. The magnitude of trait variation underlying diversity effects is usually greater among species than among genotypes within a given species, so plant species diversity effects on resource use by producers as well as on higher trophic levels should be stronger than genotypic diversity effects. Here we compared the relative importance of plant genotypic and species diversity on anti-herbivore defenses and whether such effects are mediated indirectly via diversity effects on plant growth and/or herbivore damage. To this end, we performed a large-scale field experiment where we manipulated genotypic diversity of big-leaf mahogany (*Swietenia macrophylla*) and tree species diversity, and measured effects on mahogany growth, damage by the stem-boring specialist caterpillar *Hypsipyla grandella*, and defensive traits (polyphenolics and condensed tannins in stem and leaves). We found that both forms of plant diversity had positive effects on stem (but not leaf) defenses. However, neither source of diversity influenced mahogany growth, and diversity effects on defenses were not mediated by either growth-defense trade-offs or changes in stem-borer damage. Although the mechanism(s) of diversity effects on plant defenses are yet to be determined, our study is one of the few to test for and show producer diversity effects on plant chemical defenses.

## Introduction

Ecological research conducted over the last decade has shown that plant intra- and inter-specific diversity have large effects on ecosystem processes, such as decomposition rates and productivity [1]–[6], as well as on the structure of associated communities of consumers [1]–[3], [7]–[11]. Specifically, numerous studies have found that plant diversity increases plant biomass production due to niche partitioning and more efficient resource use among species or genotypes within a given species (i.e., complementarity effect) [12], [13]. Such increases in plant biomass, as well as greater habitat complexity may in turn cascade up to influence consumers, particularly in the case of arthropods associated with plant canopies [1], [2], [8], [14], [15].

Although plant diversity effects on plant biomass (via resource use) and consumers are well-documented, little information is available about the effects of plant diversity on anti-herbivore defenses. Diversity effects on plant defenses are extremely important because they might influence herbivory and explain over-yielding [7], alter community structure at higher trophic levels (e.g. via effects on herbivores) [16], as well as mediate ecosystem processes (e.g. food web dynamics, decomposition) [17].

There are two possible mechanisms by which producer diversity may influence plant defenses. First, plant diversity is known to increase plant growth via more efficient resource use [12]; assuming that the production of anti-herbivore defenses is costly for plants [18]–[20], then greater plant growth at high diversity may lead to reduced investment in defenses via growth-defense trade-offs. To date, only one study has tested this hypothesis (indirectly) and found a trade-off between complementarity for increased plant productivity and resistance to herbivory at high diversity [21], suggesting that growth-defense trade-offs may arise due to greater allocation to plant growth. Second, positive effects of plant diversity on producer biomass frequently lead to increased herbivore loads [1], [2], [8], [14], [15] and damage [22], [23], which in turn might lead to greater investment in plant defenses. Alternatively, high diversity might lead to reduced herbivore abundance (and damage) due to mechanisms of associational resistance such as reduction in host plant density (i.e. resource concentration effects) [24], [25], and in turn reduced investment in plant defenses. This latter mechanism is predicted for specialist insect herbivores which are more sensitive to changes in the density of specific host plants [26], [27].

Despite these appealing predictions, few studies have directly evaluated the effects of plant diversity on anti-herbivore defenses [8], [28] and the previously described mechanisms for such effects have not been tested. For example, Mraja and colleagues [28] found mixed evidence for diversity effects on plant defenses as *Plantago lanceolata* plants growing in patches of high species diversity exhibited a lower concentration of foliar aucubin and total iridoid glycoside, but a greater concentration of catalpol, another important defensive compound. In addition, Moreira and colleagues [8] found that host-pine species diversity increased pine growth and herbivore density but did not significantly affect the concentration of chemical defenses in pine seedlings. However, in this work the presence of predatory ants (which were more abundant in diverse patches) may have resulted in (indirect) defense against herbivores and this could have influenced patterns of allocation to chemical defenses by plants across levels of diversity [8].

Importantly, the mechanisms by which producer diversity influences plant defenses may vary depending on the source of plant diversity. For instance, the magnitude of trait variation underlying diversity effects is usually greater among species than among genotypes within a given species, therefore we would expect that plant species diversity effects on resource use by producers as well as on higher trophic levels should be stronger than genotypic diversity effects [14], [29]; but see Crawford & Rudgers [30] as counter-example. Accordingly, greater trait variation among plant species would be expected to lead to increased niche partitioning and stronger effects on plant growth (relative to genotypic diversity effects), with this in turn causing a stronger reduction in plant defenses via growth-defense trade-offs. Alternatively, greater trait variation among plant species may lead to stronger positive (via greater plant biomass) [1], [14], [15], [22] or negative (e.g. via decreased host plant density or apparency) [24], [31], [32] effects on herbivore abundance and damage (as compared to genotypic diversity), and thus stronger effects on plant defenses compared to effects of plant genotypic diversity. Nonetheless, these predictions and their mechanisms have not been tested yet as plant intra- and inter-specific diversity effects have usually been studied separately.

**Figure 1:**
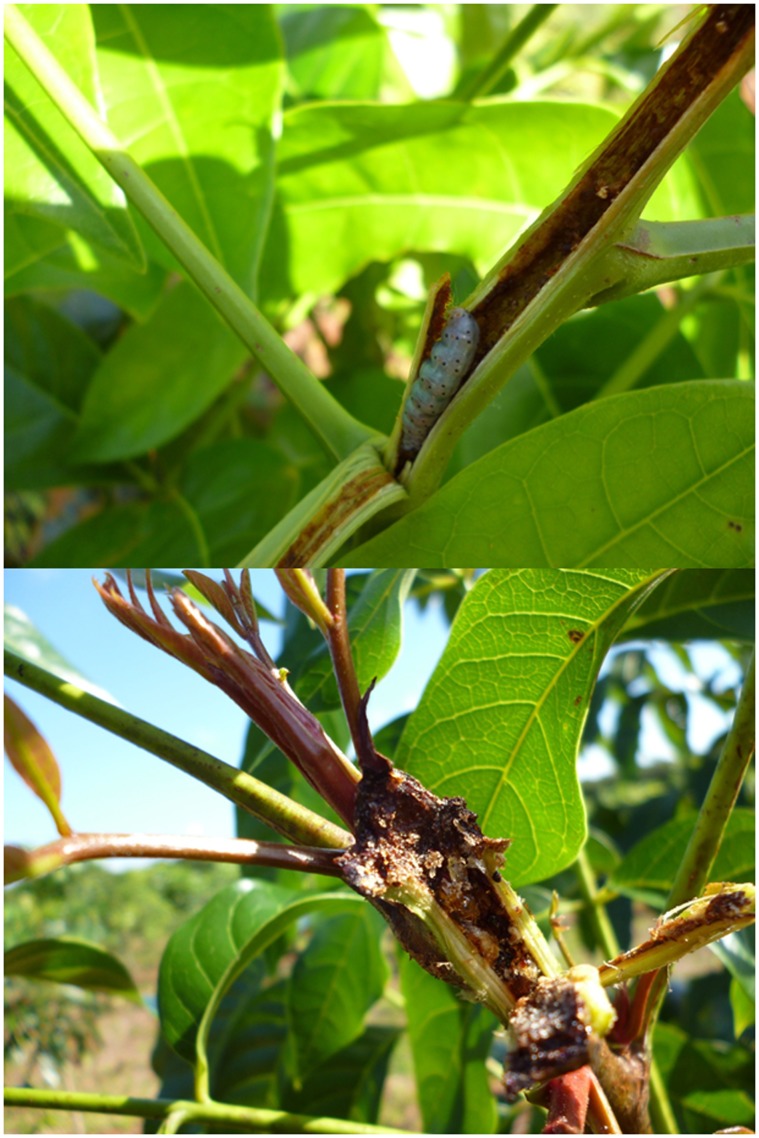
*Hypsipyla grandella* damage. Damage caused by *Hypsipyla grandella* (Lepidoptera: Pyralidae), a stem-boring caterpillar specializing on tree species of the neotropical family Meliaceae. The images show a fourth-instar larva inside a terminal shoot of a big-leaf mahogany (*Swietenia macrophylla* King, Meliaceae) sapling and the damage caused. Photo credits: Luis Abdala-Roberts.

The aim of this study was to evaluate the effects of plant species and genotypic diversity on producer anti-herbivore defenses. In particular, we were interested in (a) comparing the relative importance of these two source of plant diversity (following the prediction of stronger effects of species diversity), and (b) evaluating the mechanisms for such effects, namely if diversity effects were mediated via changes in plant growth (due to underlying growth-defense trade-offs) or herbivore damage. To address this, we performed a large-scale field experiment where we manipulated genotypic diversity of the tropical tree big-leaf mahogany (*Swietenia macrophylla*) as well as tree species diversity, and evaluated the effects of diversity on mahogany growth (total height), herbivore damage (caused by the specialist insect *Hypsipyla grandella*), and chemical defensive traits (polyphenolics and condensed tannins in stem and leaves). We focused on *H. grandella* because it is the most important herbivore (in terms of abundance and amount of damage inflicted) of mahogany in our system (Abdala-Roberts et al., unpublished data). Larvae of this herbivore carve tunnels through the terminal shoots of juvenile plants (Fig. 1) which in turn result in die-off of large portions of the plant, stem deformation, and reduced growth [33]. Overall, the present study is one of the few to test for plant diversity effects on anti-herbivore defenses, and uniquely compares such effects among sources of diversity while addressing the mechanisms for such effects. In so doing, we move beyond studying the effects of plant diversity on resource use to understanding how diversity influences plant secondary chemistry, an important but largely ignored suite of plant traits influencing herbivores and associated food webs.

## Materials and Methods

### Ethics Statement

The research did not involve manipulations of humans or animals. No specific permissions were required for our field work. The plant material used for this study was only sampled at a very limited scale and therefore had negligible effects on broader ecosystem functioning. The location is not privately-owned or protected in any way. The field studies did not involve endangered or protected species.

### Natural history

Big-leaf mahogany (*Swietenia macrophylla* King, Meliaceae) is a large-statured tree with a patchy distribution throughout much of southern Mexico, Central America, and South America [34]. In forests, big-leaf mahogany is fed upon by a suite generalist and specialist insect leaf chewers [35], [36], leaf-miners [37], and rodents [38]. The most relevant herbivore attacking mahogany in forest plantations, and particularly at our study site (Abdala-Roberts et al., unpublished data), is *H. grandella* (Lepidoptera: Pyralidae) [37], [39], a specialist stem-boring caterpillar that feeds only on a few species of Meliaceae (Fig. 1). Larvae of this herbivore carve tunnels through the terminal shoots and poles of saplings and juvenile plants (Fig. 1), resulting in severe deformations of the main stem and shunted growth [33]. Because this herbivore is a stem feeder, we expected a stronger link between damage and chemical defenses in the stem relative to defenses in other types of tissue (e.g. leaves).

In tropical forests of the Yucatan Peninsula, big-leaf mahogany co-occurs with five other tree species [40] that are the subject of this experiment, namely: *Tabebuia rosea* (Bertol.) DC. (Bignonaceae), *Ceiba pentandra* (L.) Gaertn. (Malvaceae), *Enterolobium cyclocarpum* (Jacq.) Griseb. (Fabaceae), *Piscidia piscipula* (L.) Sarg. (Fabaceae), and *Cordia dodecandra* A. DC. (Boraginaceae). These species are long-lived, deciduous, and adult trees reach from 20 m (*P. piscipula*) to 40 m (*C. pentandra*) [34], and are distributed from central México to Central and South America [34].

### Experimental design

In December 2011, we established 74 plots of 21×21 m each at a planting density of 64 plants per plot, 3 m spacing among trees, for a total of 4780 plants. Isles between plots were 6-m wide, and the experiment covered a total area of 7.2 ha Mahogany was planted in 59 of these plots which were classified into four types, depending on the diversity treatment combination: a) mahogany monocultures of one genotype (12 plots, two replicate plots/genotype; hereafter “genotypic monocultures”), b) mahogany monocultures of four genotypes (20 plots; hereafter “genotypic polycultures”), c) polycultures of four species within which all mahogany saplings were of one genotype (12 plots, two plots/genotype), and d) polycultures of four species within which mahogany plants were represented by four genotypes (15 plots). For these four plot types, treatments of both species and genotypic diversity included equal numbers of individuals of four species (one of which was always mahogany) and four mahogany genotypes drawn from pools of six species or six genotypes, respectively. All non-mahogany species were equally represented across polycultures (each species present in six polyculture plots). Likewise, mahogany genotypes were represented in a similar number of mahogany monocultures of four genotypes (8–9 plots per genotype), and also in a similar number of species polycultures where mahogany plants were of four genotypes (9–10 plots per genotype).

In this study, we sampled a subset of these 59 plots where mahogany was present (see following two sections) and within these plots we restricted our sampling only to mahogany.

### Seed sources and measurements of plant growth and herbivory

For measurements of growth and stem borer damage, we randomly selected eight mahogany plants allocated across plot types as follows (N  = 352 plants, 44 plots): 12 genotypic monoculture plots, 20 genotypic polyculture plots, and 12 species polyculture plots where mahogany was represented by one genotype; we did not sample species polyculture plots with four genotypes. From January 2011 to March 2011, we collected seeds of each tree species from adult plants located in southern Quintana Roo (SE México), and germinated at the INIFAP (Instituto Nacional de Investigaciones Forestales Agrícolas y Pecuarias) in Mocochá (21°06′N, 89°26′W, Yucatan, SE México). For all species, we collected seed from six mother trees, and distance among trees ranged from 0.5 to 50 km. In the case of mahogany, distance among mother trees ranged from 3 to 50 km which is within the distance range used by previous studies to define genetically distinct populations of this species [41], [42]. In December 2011, we established the experiment near the locality of Muna (20°24′44′′N, 89°45′13′′W, Yucatan, SE Mexico) by planting four-month old seedlings. After planting, saplings were fertilized in January 2012 with N, P, K (20∶30:10) and irrigated with 2 l of water three times per week from January 2012 until June 2012, and from January 2013 until June 2013. The rainy season typically spans from June until October and therefore artificial irrigation was unnecessary during this time period.

In late July 2013, we recorded mahogany growth by measuring total height, as well as *H. grandella* damage by examining the apical and axilar meristems of plants in search of attack sites (easily identified by the presence of frass) [43] and recorded the number of *H. grandella* attacks per plant.

### Chemical analyses of plant anti-herbivore defenses

As a proxy of quantitative chemical defences, we measured polyphenolics and condensed tannins in stems and leaves. In late July 2013, we randomly sampled four out of the eight previously mentioned mahogany plants sampled per plot for growth and stem borer attack (N  = 144 plants, 36 plots), allocated across plot types as follows: 12 (i.e. all) genotypic monocultures, 12 (out of the 20) genotypic polycultures, and 12 (i.e. all) species polycultures; again, we did not sample polycultures with four mahogany genotypes. Polyphenolics are carbon-based compounds, non-nutritious, and unpalatable for herbivores because they inhibit herbivore digestion by binding to consumed plant proteins [44]. Condensed tannins are one of the most abundant phenolic compounds in plant tissues and have strong negative effects on food digestibility and herbivore performance [44], [45]. Both defensive traits are generally recognized as herbivore feeding deterrents [44], [46], and have been shown to reduce *H. grandella* larval performance and survival [47], [48]. For each plant, we collected a 10 cm-long segment of the stem and three fully expanded, undamaged terminal leaves of one branch. Samples were transported to the laboratory on ice, immediately weighed, oven-dried (45°C to constant weight), and subsequently ground manually in a mortar with liquid nitrogen for subsequent determination of polyphenolic and condensed tannin concentration.

Polyphenolics were extracted and analysed as described by Moreira et al. [49]. Briefly, polyphenolics were extracted from 300 mg of plant tissue with aqueous methanol (1∶1 vol:vol) in an ultrasonic bath for 15 min, followed by centrifugation and subsequent dilution of the methanolic extract. Polyphenolic concentration was determined colorimetrically by the Folin-Ciocalteu method in a Biorad 650 microplate reader (Bio-Rad Laboratories Inc., Philadelphia, PA, USA) at 740 nm, using tannic acid as standard, and concentrations were based on dry weights (d.w.).

Condensed tannins were determined by the acid butanol method [50] in the same 50% aqueous methanol extract used for polyphenolics. A mixture of an aliquot of methanol extract and acid butanol (950 ml of n-butanol mixed with 50 ml of concentrated HCl) and iron (0.5 g of 2% ferric ammonium sulphate in 2N HCl) reagents was placed in a boiling water bath for 50 min and then cooled rapidly to 0°C on ice. Condensed tannins were determined colorimetrically in a Biorad 650 microplate reader at 550 nm using a commercial quebracho tannin extract (72.0% condensed tannins) as standard [51].

### Statistical analyses

We performed general linear mixed models to test for plant diversity effects on plant growth, herbivore damage (number of *H. grandella* attacks per plant), and chemical defenses (polyphenolics and condensed tannins, separately for leaves and stems). For each response variable, we performed two separate models: First, to test the effect of mahogany genotype diversity, we compared genotypic monocultures and genotypic polycultures, and second, to test for a species diversity effect, we compared genotypic monocultures and species polycultures (hereafter “Model 0” in each case). Then, we departed from these initial models and constructed “mechanistic” models which included additional covariates aimed at testing if each source of diversity influenced mahogany defenses via effects on mahogany growth and *H. grandella* attack: (i) “Model 1” tested an effect of genotypic or species diversity on mahogany defenses via trade-offs between growth and defense by including plant height as a covariate. If diversity effects on defenses are mediated by growth-defense trade-offs via increased growth, then a significant effect of diversity on defenses should become non-significant once plant size is accounted for in the model; (ii) “Model 2” tested an effect of genotypic or species diversity via changes in herbivory by including *H. grandella* attack as a covariate in the model. If diversity effects on defenses are mediated by higher or lower attack of *H. grandella* at high diversity, then a significant effect of diversity on defenses should become non-significant once herbivory is accounted for in the model. Normality was met in all cases, and we report least square means ± standard errors as descriptive statistics. All models were performed with PROC MIXED in SAS (SAS 9.2 System, SAS, Cary, NC) using plot and genotype as random effects. The former accounted for non-independence of plants sampled from the same plot, while the latter controlled for variation in growth and defenses among maternal sources. We also performed models using plot as the level of replication (results not shown) and all results were qualitatively idential.

## Results

### Consequences of plant genotypic and species diversity on mahogany growth and herbivory

**Figure 2:**
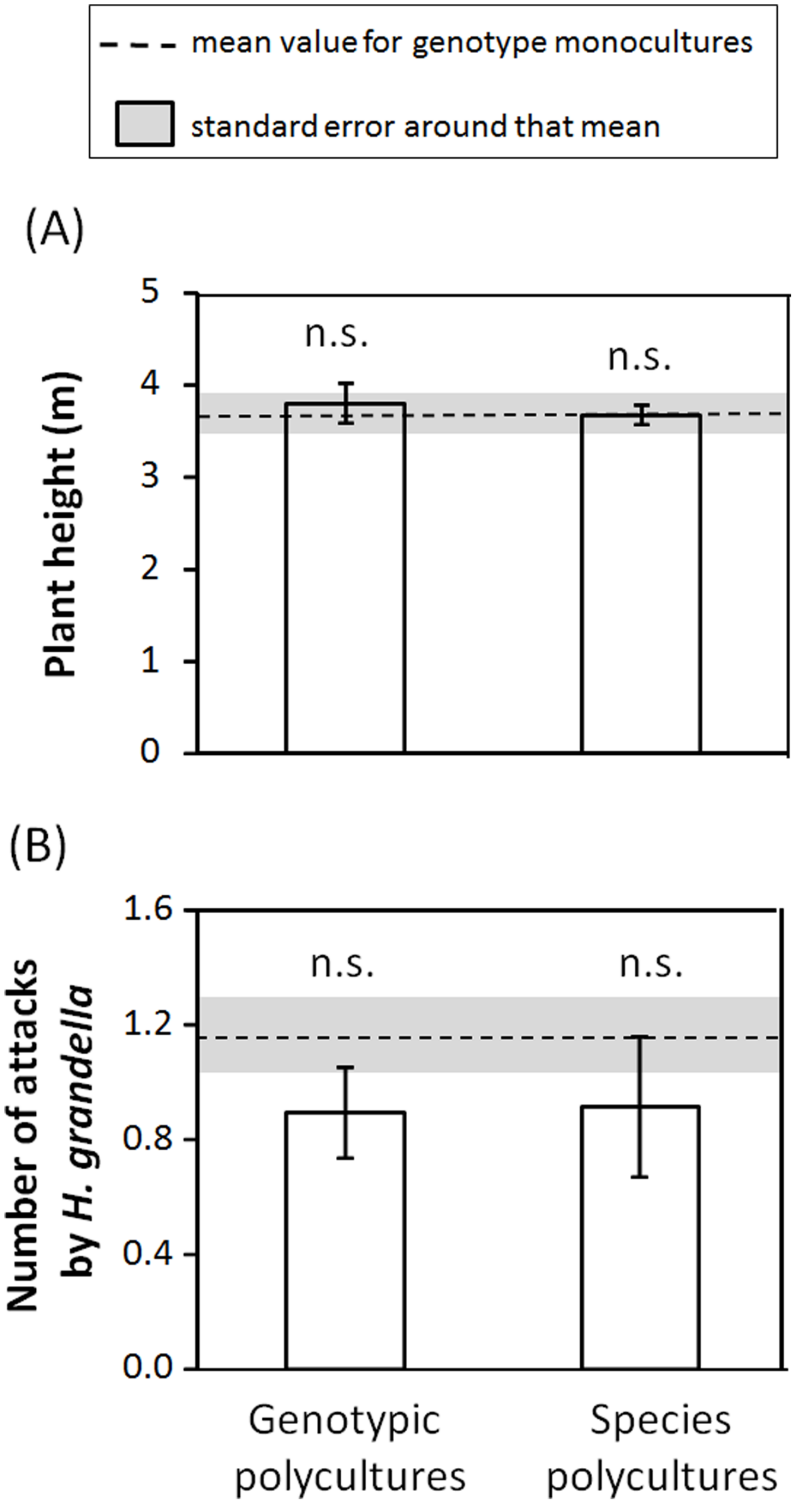
Diversity effect on growth and herbivore damage. Effect of mahogany genotypic and tree species diversity on: (A) mahogany sapling height and (B) the mean number of attack sites per plant by the specialist stem-boring insect *Hypsipyla grandella*. The dashed line represents the mean value for genotype monocultures (N  = 12) and the shaded area represents the standard error around that mean. Least-square means ± S.E. (N  = 20 genotypic polycultures and N  = 12 species polycultures). “(n.s.)” in the figures indicates non-significant differences (*P*<0.05) between a given diversity treatment and the genotypic monoculture treatment.

Eighteen months after planting, the mean size of mahogany plants was 3.65±0.11 m, 3.79±0.09 m, and 3.67±0.11 m for genotypic monocultures, genotypic polycultures, and species polycultures, respectively. We found that neither genotypic diversity nor species diversity had significant effects on plant height (Table 1, Fig. 2A). On the other hand, we found that 48% of all mahogany saplings were attacked by *H. grandella*. Although attack was lower in genotypic and species polycultures (30% in both cases) relative to genotypic monocultures, we were not able to detect a significant effect of either source of plant diversity on attack by this herbivore (Table 1, Fig. 2B).

**Table 1:** Diversity effect on growth and herbivore damage.

	Plant height		Damage by *H. grandella*	
**a) Genotypic diversity**	F_1,218_	*P*	F_1,218_	*P*
Diversity	1.03	0.311	1.47	0.226
**b) Species diversity**	F_1,166_	*P*	F_1,167_	*P*
Diversity	0.04	0.849	0.90	0.344

Summary of results from generalized linear mixed models testing for the effects of (a) big-leaf mahogany (*Swietenia macrophylla* King, Meliaceae) genotypic diversity and (b) tree species diversity on mahogany growth and damage by the specialist *Hypsipyla grandella* (number of attack sites per plant). We tested the effect of mahogany genotypic diversity by comparing genotypic monocultures to genotypic polycultures, whereas to test for a species diversity effect we compared genotypic monocultures and species polycultures. F-values and associated significance levels (*P*) are shown, as well as numerator and denominator degrees of freedom (subscripts).

### Consequences of plant genotypic and species diversity on mahogany defenses

**Figure 3:**
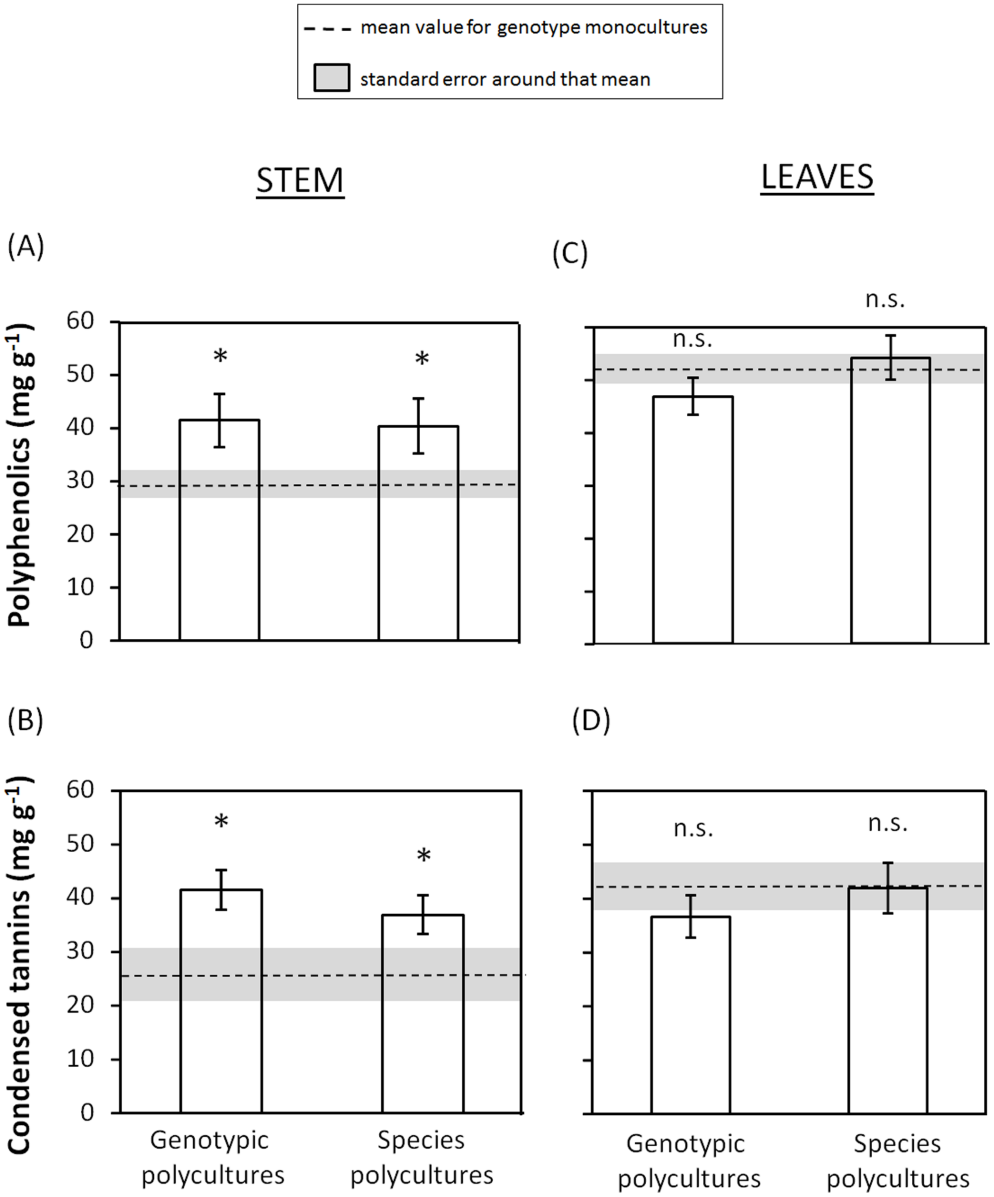
Diversity effect on plant defenses. Effect of mahogany genotypic and species diversity on the concentration of: (A) mahogany stem polyphenolics, (B) stem tannins, (C) leaf polyphenolics, and (D) leaf tannins. The dashed line represents the mean value for mahogany genotypic monocultures (N  = 12) and the shaded area represents the standard error around that mean. Least-square means ± S.E. (N  = 12 for genotypic polycultures and N  = 12 for species polycultures). Significant differences (*P*<0.05) between a given diversity treatment and the genotypic monoculture treatment are indicated by an asterisk.

Genotypic diversity had a significant effect on the concentration of chemical defenses in stems (Model 0, Table 2a). Specifically, we found that the concentration of polyphenolics and condensed tannins in stems were 40% and 60% greater, respectively, in genotypic polycultures than in genotypic monocultures (Fig. 3A, 3B). For both types of defenses, this positive effect of genotypic diversity remained significant after accounting for *H. grandella* attack (Model 1, Table 2a). Similarly, the genotypic diversity effect on stem polyphenolics and tannins remained significant after accounting for plant height (Model 2, Table 2a). Herbivore attack had only significant effects on stem tannins (Model 2, Table 2a), whereas plant size had no significant effects on stem defenses (Model 1, Table 2a). Similarly, we found that species diversity also had a significant effect on stem defenses (Model 0, Table 2b), with the concentration of polyphenolics and condensed tannins in stems being 36% and 42% greater, respectively, in species polycultures compared with genotypic monocultures (Fig. 3A, 3B). Such effect remained significant after accounting for plant height (Model 1, Table 2b) or *H. grandella* attack (Model 2, Table 2b). Plant height and herbivory damage effects were non-significant in these models (Models 1 and 2, Table 2a, 2b), suggesting that Model 0 is the most appropriate.

**Table 2:** Diversity effect on plant defenses.

	Stem phenolics		Leaf phenolics		Stem tannins		Leaf tannins	
a) Genotypic diversity	F	*P*	F	*P*	F	*P*	F	*P*
*Model 0*								
Diversity ^(1,63)^	4.33	**0.041**	2.07	0.155	5.04	**0.028**	0.94	0.335
*Model 1*								
Diversity ^(1,62)^	4.16	**0.046**	1.99	0.164	4.44	**0.039**	0.98	0.326
Height ^(1,62)^	0.00	0.978	0.00	0.980	0.42	0.520	0.06	0.813
*Model 2*								
Diversity ^(1,62)^	4.39	**0.040**	2.06	0.156	5.21	**0.026**	0.93	0.338
Damage by *H.grandella* ^(1,62)^	2.60	0.112	0.52	0.473	4.37	**0.041**	0.03	0.873
**b) Species diversity**	**F**	***P***	**F**	***P***	**F**	***P***	**F**	***P***
*Model 0*								
Diversity ^(1,67)^	4.16	**0.045**	0.03	0.874	4.27	**0.043**	0.04	0.841
*Model 1*								
Diversity ^(1,66)^	4.07	**0.048**	0.04	0.876	4.32	**0.042**	0.04	0.843
Height ^(1,66)^	0.05	0.819	0.04	0.847	0.19	0.668	0.00	0.945
*Model 2*								
Diversity ^(1,66)^	4.20	**0.044**	0.03	0.852	4.15	**0.046**	0.06	0.801
Damage by *H.grandella* ^(1,66)^	0.13	0.721	0.34	0.563	0.29	0.590	1.17	0.283

Summary of results from generalized linear mixed models testing for the effects of (a) big-leaf mahogany genotypic diversity and (b) plant species diversity on mahogany chemical defenses (polyphenolics and condensed tannins in the stem and leaves). To test the effect of mahogany genotype diversity we compared genotypic monocultures and genotypic polycultures, whereas to test for a species diversity effect we compared genotypic monocultures and species polycultures. Initial models testing only for an effect of diversity are designed as “Model 0”. For models labelled “Model 1”, we tested for an effect of diversity on defenses via growth-defense trade-offs by including plant height (proxy of growth) as a covariate. Finally, for models labelled “Model 2” we tested an effect of diversity via changes in herbivory by including *H. grandella* attack as a covariate. F-values and associated significance levels (*P*) are shown, as well as degrees of freedom (as subscripts in parenthesis). Significant (*P*<0.05) and marginal (0.05<*P*<0.10) *P* values are typed in bold.

By contrast, genotypic and species diversity did not have significant effects on the concentration of polyphenolics or condensed tannins in leaves (Model 0, Tables 2a, 2b; Fig. 3C, 3D). The effect of plant genotypic and species diversity on leaf polyphenolics and condensed tannins remained non-significant after accounting for plant height (Model 1, Tables 2a, 2b) or *H. grandella* attack (Model 2, Tables 2a, 2b), and neither of these covariates influenced leaf defensive traits.

## Discussion

Our results showed that plant genotypic and species diversity had strong positive effects on stem (but not leaf) anti-herbivore defenses in big-leaf mahogany, namely the concentration of stem polyphenolics and condensed tannins. In addition, the effects of these two sources of plant diversity on defenses were similar in magnitude. Contrarily to our expectations, positive effects from both sources of plant diversity were not mediated by the effects of diversity on plant growth or herbivore damage. Although the mechanism(s) of diversity effects on plant defenses in this study are yet to be determined, our study is one of the few to test for and show producer diversity effects on plant chemical defenses (i.e. secondary compounds). Such effects were particularly strong and are likely to play an important role in mediating plant diversity effects on higher trophic levels and ecosystem function.

To the best of our knowledge, there are no previous studies testing the relative contribution of plant intra- and inter-specific diversity on plant defense allocation patterns. Our results showed that plant genotypic and species diversity effects on mahogany anti-herbivore defenses tended to be similar in magnitude, despite the presumption that greater magnitude of trait variation among species than among genotypes should lead to stronger effects of species diversity (relative to genotypic diversity) on producers. Moreover, for stem tannins we even observed a stronger effect of genotypic diversity relative to species diversity. Accordingly, these findings agree with recent work showing that plant intra- and inter-specific diversity effects on ecosystem function and arthropod communities can be of similar importance [2], [14], [52]. For example, Crawford & Rudgers [52] found effects of similar magnitude from species and genotypic diversity on biomass in *Ammophila breviligulata*, a dominant species in dune ecosystems. Similarly, Cook-Patton et al. [14] found equivalent increases in aboveground biomass and arthropod species richness due to genotypic and species diversity for the common evening primrose (*Oenothera biennis*). Nonetheless, further research in other systems comparing the effects of plant intra- and inter-specific diversity is necessary in order to assess the relative importance and mechanisms by which different forms of plant diversity shape anti-herbivore defenses in plants, and in turn potentially explain differential effects on arthropod faunas.

Our finding that diversity did not influence plant defenses via growth-defense trade-offs is not surprising, given that neither source of diversity influenced mahogany growth. Contrary to our findings, results from a previous study by McArt & Thaler [21] are consistent with the idea of growth-defense trade-offs act as a mechanism of diversity effects on plant defenses. Specifically, they found a trade-off where increased productivity of *Oenothera biennis* (via complementarity effects) at high genotypic diversity resulted in reduced resistance to an exotic leaf herbivore [21]. In addition, our results also run counter with the idea that producer diversity effects on plant defenses are mediated through effects on herbivores. In this case, a previous study by Mraja et al. [28] agrees with our findings, as they found that diversity effects on *P. lanceolata* were only weakly related to changes in herbivory across levels of diversity. Our results showed a tendency for a negative effect of plant diversity on *H. grandella* attack, suggesting that some unknown mechanism drove an increase in plant defenses, and that such effect in turn could have negatively influenced stem borer attack. However, this argument remains speculative as reductions in attack by this specialist herbivore may have also responded to resource concentration effects with increasing genotypic or species diversity, as suggested by previous findings in this system (Abdala-Roberts et al. unpublished data). Regardless of the mechanism at work, evidence for diversity effects on plant defenses remains limited and further research is needed to derive general patterns and determine which mechanisms and defensive compounds are more important and under what conditions.

Although we found that plant diversity effects on anti-herbivore defenses were not mediated by diversity effects on *H. grandella* attack or via growth-defense trade-offs, these mechanisms cannot be entirely discarded. First, it is possible that our measure of *H. grandella* abundance (i.e. number of attack sites per plant) was not predictive of the amount of damage (and thus defense induction) experienced by each plant. Second, and related also to the effects of diversity on herbivory, previous work in our system (during 2012) has shown that attack by *H. grandella* was significantly lower in species polycultures at the middle of the rainy season (early September), but this pattern reversed towards the end of the rainy season (late October) (Abdala-Roberts et al., unpublished data). Therefore, whereas we associated plant defenses with current patterns of herbivory (tissue samples taken during the same month as herbivore measurements), it is possible that stem-borer attack levels during previous months would have been a better predictor of defensive investment. Third, although results suggest that diversity effects on defenses via growth-defense trade-offs are not occurring at present in this system, our measurements were conducted at an early time point in the experiment. Accordingly, it is possible that such trade-offs may arise subsequently, once diversity effects on plant growth presumably become stronger [13], [53].

It is important to note that some authors have suggested that producer diversity may influence plant defenses in the absence of effects on plant growth and growth-defense trade-offs [28], [54], [55]. For example, plant species diversity may decrease light availability (due to architecture differences among species or genotypes) and such effect is predicted to reduce the concentration of carbon-based defenses such as phenolics, which are involved in photo-protection [56]. Accordingly, we previously found that low light availability reduces the concentration of polyphenolics and tannins in leaves and stems of mahogany [57]. However, in the present study we instead found a positive effect of species diversity on defenses which runs counter the argument that diversity effects on polyphenolics and tannins were mediated by light availability. Alternatively, as suggested by other studies, it is possible that plant species diversity improves nutrient acquisition (e.g. nitrogen) and use by plants which in turn increased nutrient concentrations in tissues [28], [55], [58] as well as the concentration of nitrogen-based defenses [28]. Finally, there is evidence that plants are able to recognize conspecific vs. hetero-specific individuals, as well as more closely vs. more distantly related individuals of their species and modulate the release of volatiles associated with defense [59]. Based on this, the prediction would be increased expression of defenses in monocultures (species or genotypic) as there would be a greater density of con-specifics or individuals of the same genotype emitting volatiles. However, we found the opposite pattern, which suggests that this mechanism was not at work.

In summary, the results from this study demonstrated that both plant inter- and intra-specific diversity can cause important changes in allocation to chemical defenses by plants, but that such effects were not driven by changes in plant growth (and thus growth-defense trade-offs) or herbivore damage. We suggest that additional abiotic factors (e.g. nutrient availability and uptake) and mechanisms should be considered in future studies in order to fully understand the observed patterns. Understanding the mechanisms by which plant diversity influences traits of importance to herbivores, in particular plant secondary metabolites, will contribute to a better understanding of plant diversity effects on consumers and ecosystem function.
